# Transcriptional activation of fucosyltransferase (FUT) genes using the CRISPR-dCas9-VPR technology reveals potent *N*-glycome alterations in colorectal cancer cells

**DOI:** 10.1093/glycob/cwy096

**Published:** 2018-10-22

**Authors:** Athanasios Blanas, Lenneke A M Cornelissen, Maximilianos Kotsias, Joost C van der Horst, Henri J van de Vrugt, Hakan Kalay, Daniel I R Spencer, Rad P Kozak, Sandra J van Vliet

**Affiliations:** 1 Amsterdam UMC, Vrije Universiteit Amsterdam, Department of Molecular Cell Biology and Immunology, Cancer Center Amsterdam, Amsterdam Infection & Immunity Institute, HZ Amsterdam, the Netherlands; 2 Ludger Ltd, Culham Science Centre, Abingdon, United Kingdom; 3 Amsterdam UMC, Vrije Universiteit Amsterdam, Oncogenetics, Department of Clinical Genetics, Cancer Center Amsterdam, HV Amsterdam, the Netherlands

**Keywords:** colorectal cancer, CRISPR-dCas9-VPR, fucosylation, gene activation, *N*-glycans

## Abstract

Aberrant fucosylation in cancer cells is considered as a signature of malignant cell transformation and it is associated with tumor progression, metastasis and resistance to chemotherapy. Specifically, in colorectal cancer cells, increased levels of the fucosylated Lewis^x^ antigen are attributed to the deregulated expression of pertinent fucosyltransferases, like fucosyltransferase 4 (FUT4) and fucosyltransferase 9 (FUT9). However, the lack of experimental models closely mimicking cancer-specific regulation of fucosyltransferase gene expression has, so far, limited our knowledge regarding the substrate specificity of these enzymes and the impact of Lewis^x^ synthesis on the glycome of colorectal cancer cells. Therefore, we sought to transcriptionally activate the *Fut4* and *Fut9* genes in the well-known murine colorectal cancer cell line, MC38, which lacks expression of the FUT4 and FUT9 enzymes. For this purpose, we utilized a physiologically relevant, guide RNA-based model of de novo gene expression, namely the CRISPR-dCas9-VPR system. Induction of the *Fut4* and *Fut9* genes in MC38 cells using CRISPR-dCas9-VPR resulted in specific neo-expression of functional Lewis^x^ antigen on the cell surface. Interestingly, Lewis^x^ was mainly carried by *N*-linked glycans in both MC38-FUT4 and MC38-FUT9 cells, despite pronounced differences in the biosynthetic properties and the expression stability of the induced enzymes. Moreover, Lewis^x^ expression was found to influence core-fucosylation, sialylation, antennarity and the subtypes of *N*-glycans in the MC38-glycovariants. In conclusion, exploiting the CRISPR-dCas9-VPR system to augment glycosyltransferase expression is a promising method of transcriptional gene activation with broad application possibilities in glycobiology and oncology research.

## Introduction

Over the last few years, genome engineering through the clustered regularly interspaced short palindromic repeats (CRISPR)-Cas9 system has received increased attention and is gradually becoming a method of choice for studying various biological processes, including protein and lipid glycosylation. To date, the CRISPR-Cas9 technology is considered as the most precise, efficient and cost-effective gene editing tool available (Chandrasegaran and Carroll 2016).

Despite the vast complexity of the mammalian glycome, glycosyltransferases have been genetically classified as members of homologous isoenzyme families that influence glycosylation globally (Hansen et al. 2015). Therefore, different applications of the CRISPR-Cas9 system can serve as the ultimate toolbox for investigating the biological functions of glycans, through genetic abrogation of the respective biosynthetic pathways in primary cells and cell lines (Narimatsu et al. 2018). For example, the gene knock-out application of CRISPR-Cas9 has already assisted to a better understanding of how glycosylation influences human leukocyte adhesion to endothelial cells (Stolfa et al. 2016) or the transport of galectins to the cell surface (Stewart et al. 2017).

**Fig. 1.**
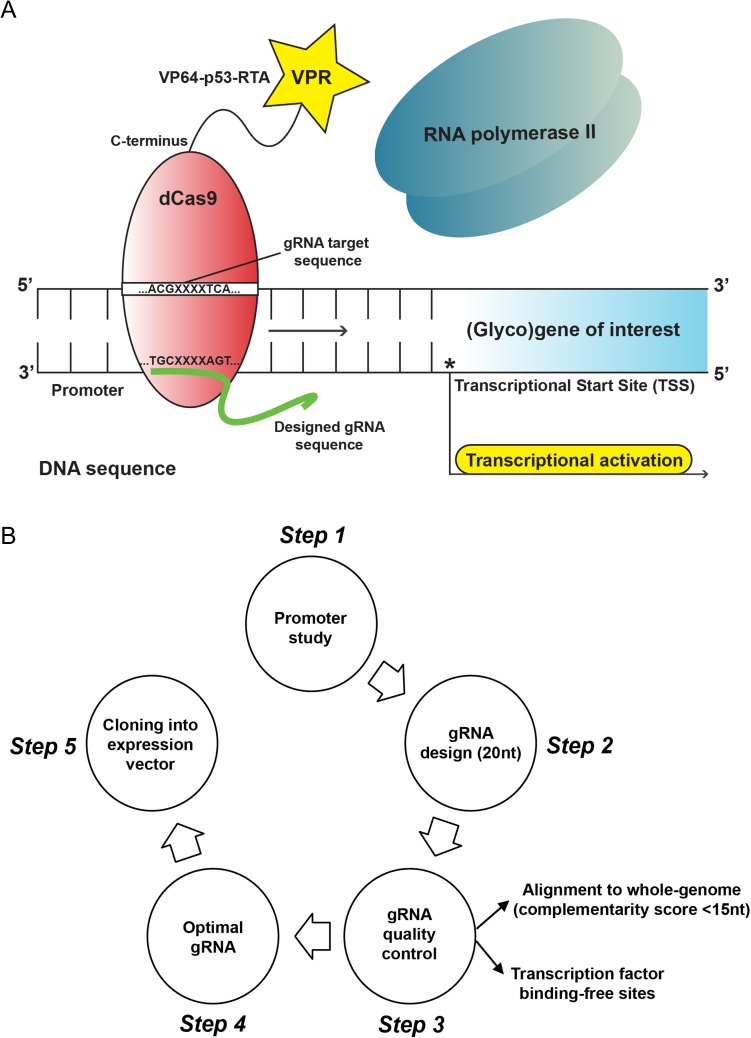
Model and experimental design for the CRISPR-dCas9-VPR system. (**A**) Principle of transcriptional gene activation using the CRISPR-dCas9-VPR technology. One or multiple guide RNA (gRNA) sequences that specifically target the complementary promoter region of the (glyco)gene of interest, result in direct recruitment of the catalytically inactive Cas9 nuclease (known as defective or deactivated Cas9) to this site. The following interaction between VPR (VP64-p65-Rta chimeric activator fused to the C-terminus of dCas9) and RNA polymerase II drives the induction of target gene expression. (**B**) Overview of the five-step experimental design applied for transcriptional activation of the murine *Fut4* and *Fut9* genes using the CRISPR-dCas9-VPR technology.

Besides the classical gene editing (e.g gene knock-out) applications, tools for successful regulation of gene expression using the CRISPR-Cas9 technology have been also introduced, such as the CRISPR-dCas9-VPR system (Chavez et al. 2015). In sharp contrast with traditional methods of gene overexpression with the use of selected cDNA clones, the CRISPR-dCas9-VPR toolkit facilitates *de novo* gene transcription that occurs physiologically within the nucleus of the cell and its native chromosomal context. In this case, one or multiple guide RNA (gRNA) sequences specifically target the promoter region of the gene of interest, resulting in direct recruitment of the catalytically inactive Cas9 nuclease (called defective or deactivated Cas9) to this site. However, a major difference compared to the CRISPR-Cas9 gene editing tools is that the dCas9 protein is now fused to a hybrid tripartite activation domain (VP64-p53-Rta), known as VPR. The subsequent interaction between the VPR activation unit of dCas9 and the RNA polymerase II and/or other transcription factors eventually drives the expression of the gene of interest (Figure 1A).

We hypothesized that induction of gene expression using the CRISPR-dCas9-VPR system could be reliably applied to glycobiology research through the efficient and specific transcriptional programming of glycosyltransferase genes. Importantly, by employing CRISPR-dCas9-VPR, all the critical regulatory mechanisms associated with glycosyltransferase gene expression can be easily unraveled, since they are still active in this model and not simply bypassed. In the past, significant changes in glycosylation due to the use of cDNA clones have been observed (van Leeuwen et al. 2006). Moreover, complex epigenetic modifications of genes involved in protein and lipid glycosylation (Zoldos et al. 2010, Lauc et al. 2014) that are often completely missed or undermined when cDNA clones are used, can be now further assessed with CRISPR-dCas9-VPR (Lo and Qi 2017). This is of utmost importance for dissecting the mechanisms that lead to an aberrant expression profile of certain glycosyltransferases under pathological conditions, as in the case of cancer.

In general, tumor cells are characterized by a tremendous change in their cell surface glycome, as a result of genetic or epigenetic alterations in the expression of particular glycosyltransferase genes. Specifically, cancer cells exhibit elevated levels of fucosylation, sialylation and branched *N*-linked glycans compared to their healthy counterparts, due to overexpression of certain genes involved in the respective biosynthetic pathways (Pinho and Reis 2015). Therefore, exploitation of the CRISPR-dCas9-VPR system for transcriptional reprogramming of genes related to cancer glycosylation could provide a better insight into the role of individual glycosyltransferases, as well as the impact of tumor-associated glycans on cancer cell biology.

One well-known tumor-associated glycan motif is the fucosylated Lewis^x^ trisaccharide (Galβ1-4(Fucα1-3)GlcNAcβ1), alternatively known as the stage-specific embryonic antigen 1 (SSEA-1) or CD15 antigen. It is a member of the human histo-blood group antigen system, broadly known as the Lewis antigen system. Overexpression of this epitope has been reported in many types of cancer, including colorectal cancer (Blanas et al. 2018). Lewis^x^ is recognized by designated C-type lectin receptors (CLRs) expressed on antigen-presenting cells, such as DC-SIGN (CD209) in humans (Appelmelk et al. 2003) and MGL-1 (CD301a) in mice (Singh et al. 2009). Although different fucosyltransferases are responsible for its synthesis, fucosyltransferase 4 (FUT4) and fucosyltransferase 9 (FUT9) are the most competent ones in synthesizing Lewis^x^ (Mondal et al. 2018). Notably, both enzymes are strongly associated with colorectal cancer progression, metastasis and resistance to chemotherapy (Giordano et al. 2015, Auslander et al. 2017). However, the biosynthetic properties of the FUT4 and FUT9 enzymes and the potential effect of increased Lewis^x^ expression on the glycosylation status of colorectal cancer cells have not been fully investigated yet.

To this end, we are the first to apply the CRISPR-dCas9-VPR targeting system to transcriptionally activate the *Fut4* and *Fut9* fucosyltransferase genes in MC38 cells, a murine colorectal adenocarcinoma cell line that is commonly used in pre-clinical mouse models for this disease (McIntyre et al. 2015, Zhao et al. 2017). Following this approach, we successfully generated FUT4- or FUT9-expressing MC38 glyco-engineered cell lines and examined changes in their respective glycosylation profiles, focusing on biosynthesis of the fucosylated Lewis^x^ determinant and its impact on the cancer cell glycome. We believe that this novel methodology of gene expression can be further applied both to human and murine glycosyltransferases involved in tumorigenesis or other disorders and thus set the framework to elucidate the exact implication of these enzymes (or their synthesized glycan structures) in different aspects of disease pathogenesis. Moreover, we consider our study as a representative example of how advances in the CRISPR technology can benefit research investigations focused on glycosylation, thus highlighting its role in health and disease.

## Results

### Design, selection and quality control of the murine *Fut4* and *Fut9* gene targeting gRNA sequences

A key factor for precise, but also efficient, gene targeting using the CRISPR-dCas9-VPR system is the design of the corresponding gRNA sequences. To date, several prediction tools have been developed for this purpose (Hsu et al. 2013, Doench et al. 2014, Heigwer et al. 2014, Montague et al. 2014), providing detailed lists of proposed gRNAs to the user. However, the final decision about the exact gRNAs that should be used remains a big challenge and a protocol for precisely narrowing down all the possible options is still missing. Therefore, we here present the workflow followed by us for the selection and quality control of the designed gRNAs targeting the murine *Fut4* and *Fut9* genes (Figure 1B). Importantly, we believe that this process can be easily adapted and utilized for the selection of gRNA sequences that specifically target any other (glyco)gene of interest.

In more detail, we initially reviewed the literature giving emphasis to the promoter regions as well as the known regulatory elements that have been implicated in determining the expression of the *Fut4* and *Fut9* fucosyltransferase genes. Then, we designed 20-nucleotide gRNAs targeting sequences in close proximity to the transcriptional start site of each gene, since transcriptional gene activation is most effective when the target sequence is within the range −400 and +100 bp of the corresponding transcriptional start site (Gilbert et al. 2014, Konermann et al. 2015, Liao et al. 2017). The gRNA sequences were designed using the E-CRISP platform (http://www.e-crisp.org/E-CRISP/) (Heigwer et al. 2014) and we carefully selected only those gRNAs that could be validated by a second prediction tool, such as the CRISPR design (http://crispr.mit.edu/) (Hsu et al. 2013). In order to exclude non-specific gRNAs with potential off-target effects from our analysis, we exploited the Basic Local Alignment Search Tool (BLAST) and further selected those ones that had the minimum complementarity score (<15) with respect to the whole-genome. Finally, by using the transcription factor binding-site prediction tool Tfsitescan (http://www.ifti.org/cgi-bin/ifti/Tfsitescan.pl), we ended up with two gRNAs for each FUT gene (Supplementary Table I), targeting transcription factor binding-free sequences in close proximity to the transcriptional start site and fulfilling the remainder of the criteria mentioned above. These gRNA sequences were considered as “optimal” according to our established gRNA quality control system and were cloned into the appropriate expression vector for the generation of the final gRNA constructs (Figure 1B).

### Transcriptional activation of the *Fut4* and *Fut9* genes leads to specific neo-expression of the Lewis^x^ antigen in MC38-glycovariants

Although high expression of fucosylated epitopes has been observed in many human colorectal cancer cell lines (Holst et al. 2016), our MC38 wild type cells did not express any fucosylated type I or type II Lewis antigens on their cell surface (Supplementary Figure 1A) and were completely devoid of *Fut4* and *Fut9* gene expression (Supplementary Figure 1B). Therefore, we considered MC38 as an ideal candidate cell line for screening the functionality of the designed *Fut4* and *Fut9* gene targeting gRNAs in vitro. Expression of Lewis^x^ was used as a collective readout for efficient induction of *Fut4* or *Fut9* gene transcription and subsequent fucosyltransferase function. Specifically, we examined the efficiency of the selected gRNA sequences by co-transfecting MC38 cells with the Sp-dCas9-VR plasmid and one of the *Fut4*- or *Fut9*-targeting gRNA constructs (Supplementary Table I). Two days after transfection, MC38 cells were harvested and stained with an anti-Lewis^x^ specific antibody (clone P12) and the expression levels of cell surface Lewis^x^ were assessed by flow cytometric analysis (Supplementary Figure 1E). Interestingly, MC38 cells that were transiently transfected with only one of the two *Fut4*-targeting gRNAs (gRNA#4-1 or gRNA#4-2) or the *Fut9*-targeting gRNAs (gRNA#9-1 or gRNA#9-2) displayed higher expression of Lewis^x^ compared to MC38 cells transfected with Sp-dCas9-VPR construct only (control). This implied that all our four selected gRNAs were functional by means of promoter targeting and that the efficiency of the CRISPR-dCas9-VPR system was successfully translated into functional glycosyltransferases. In addition, we further confirmed the functionality of the designed gRNA sequences by transiently transfecting two different murine cancer cell lines negative for *Fut4* and *Fut9* gene expression, Panc02 (pancreatic cancer) and LL/2 (Lewis Lung cancer) cells (Supplementary Figure 1C, D, F, G), following exactly the same procedure as mentioned above and again obtained *de novo* expression of Lewis^x^. The small discrepancies observed in the efficiency of Lewis^x^ induction, though, clearly indicate the sequence-specific and cell line-dependent variation of the different gRNAs used in our experiments. As gRNA#4-1 and gRNA#9-1 exhibited slightly higher targeting efficiencies in MC38 cells specifically, using Lewis^x^ expression as a final readout, we proceeded with only these two single gRNAs for the remainder of our studies.

**Fig. 2.**
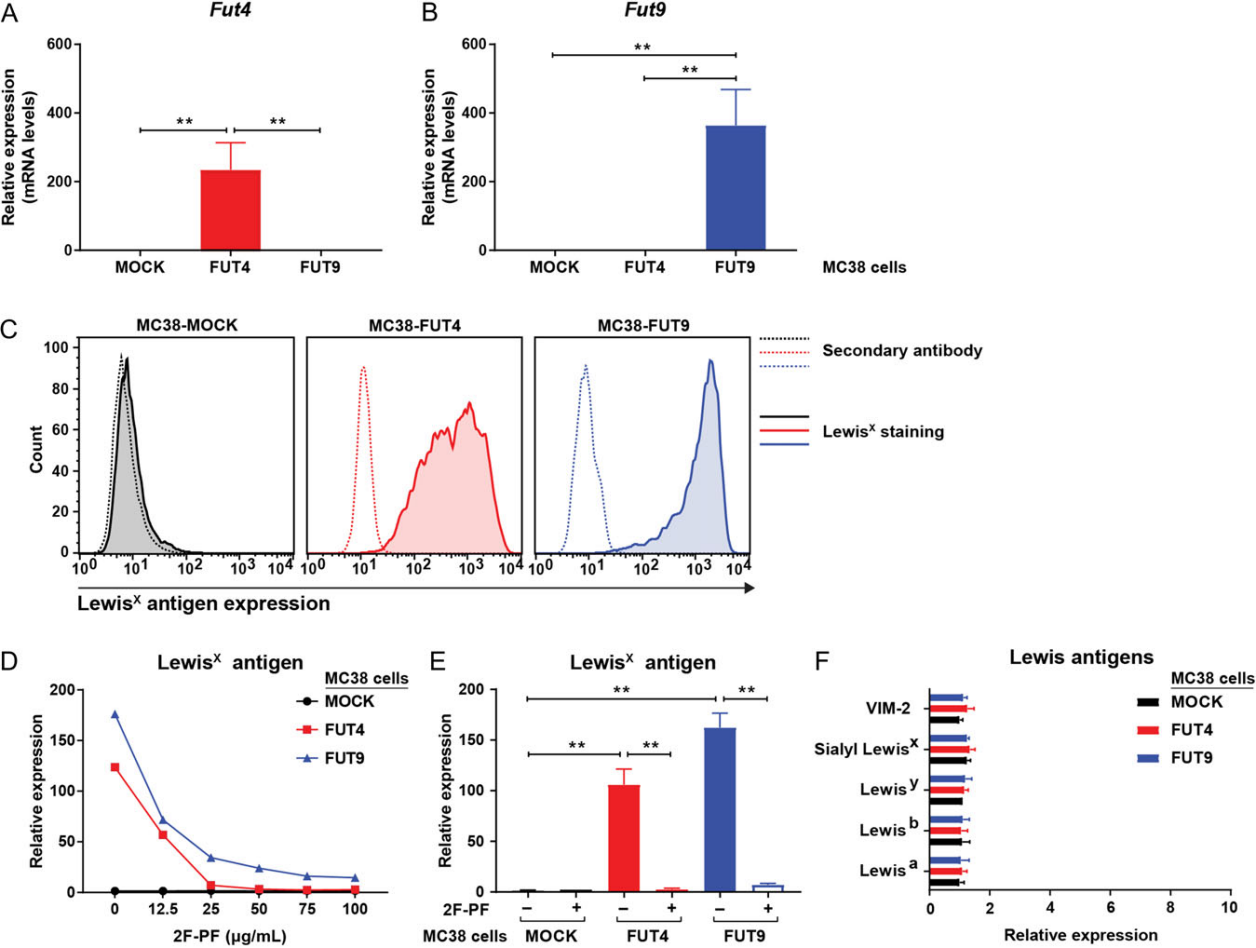
Specific neo-expression of the Lewis^x^ epitope on the surface of MC38-FUT4 and MC38-FUT9 glycovariants generated by the CRISPR-dCas9-VPR technology. (**A** and **B**) RT-PCR-based assessment of the *Fut4* and *Fut9* mRNA levels in MC38-glycovariants generated upon transcriptional gene activation using the CRISPR-dCas9-VPR system. Expression was normalized to the housekeeping gene *GAPDH* (*M. musculus*). Statistical differences relative to MC38-MOCK cells are depicted. (**C**) Flow cytometric analysis of the Lewis^x^ antigen expression on the surface of MC38 glycovariants using a mouse anti-Lewis^x^ monoclonal antibody (clone P12). Dotted lines represent staining with the secondary antibody alone, whereas solid lines represent the Lewis^x^ staining. Histograms representative of at least three independent experiments are shown. (**D**) MC38-glycovariants were cultured in vitro for 48 h with different concentrations of the fucosyltransferase inhibitor 2F-peracetyl fucose (2F-PF). Expression levels of Lewis^x^ were assessed by flow cytometry. (**E**) Expression of Lewis^x^ on the surface of MC38-FUT4 and MC38-FUT9 cells is abrogated after treatment of cells with 100 μg/mL of the fucosyltransferase inhibitor 2F-peracetyl fucose (2F-PF). Mean fluorescent intensities of three independent experiments were normalized to the binding of the secondary antibody alone. (**F**) Expression of other known type I and type II Lewis antigens (structurally related to Lewis^x^) on the cell-surface of MC38-glycovariants, as analyzed by flow cytometry. Mean fluorescent intensities of three independent experiments were normalized to the binding of the secondary antibody alone. Statistical significance was determined by a Student’s unpaired *t* test with Welch’s correction (**P < 0.01).

Our next step was to generate stable MC38-FUT4 or MC38-FUT9 expressing cells and to evaluate possible changes in their cell surface glycosylation profiles. In this case and compared to MC38-MOCK cells, we observed >200-fold induction of the *Fut4* mRNA levels in MC38-FUT4 cells (Figure 2A) and >300-fold induction of the *Fut9* mRNA levels in MC38-FUT9 cells (Figure 2B), respectively. Also, no induction of *Fut9* gene expression was observed in MC38-FUT4 cells and vice versa, thus showing the specificity of our gRNAs and the CRISPR-dCas9-VPR system. Subsequently, stably-transfected MC38 glycovariants were stained with an anti-Lewis^x^ specific antibody and the expression levels of cell surface Lewis^x^ were assessed using flow cytometry. Consistent with the increased mRNA levels of the *Fut4* and the *Fut9* genes upon gene activation, significant neo-expression of the Lewis^x^ epitope could be detected on the cell surface of both MC38-FUT4 (>100-fold induction) and MC38-FUT9 cells (>150-fold induction), but not on the MC38-MOCK cells (Figure 2C, E). These results indicate that besides transcriptional gene activation, the CRISPR-dCas9-VPR system can be directly coupled to physiologically relevant fucosyltransferase function, represented in this case by the biosynthesis and the elevated cell surface expression of the Lewis^x^ determinant in MC38-FUT4 and MC38-FUT9 glycovariants.

In order to confirm the specificity of the *de novo* Lewis^x^ expression, cells were treated with different concentrations of the fucosyltransferase inhibitor 2F-peracetyl fucose (2F-PF) (Rillahan et al. 2012) and stained with the anti-Lewis^x^ antibody. As expected, expression of Lewis^x^ gradually decreased on the surface of MC38-FUT4 and MC38-FUT9 cells upon escalating doses of 2F-PF, with a maximal effect observed at an inhibitor concentration of 100 μg/mL (Figure 2D). Noticeably, Lewis^x^ expression was totally absent on MC38-MOCK cells, irrespective of the inhibitor treatment or not. Besides Lewis^x^, no other fucosylated type I or type II Lewis antigens (structurally related to Lewis^x^) could be recorded on the surface of MC38 glycovariants (Figure 2F), confirming that transcriptional activation of the *Fut4* and *Fut9* genes in MC38 cells specifically results in increased levels of the Lewis^x^ epitope only.

During our studies, we noticed that in MC38-FUT4 cells specifically, expression of the Lewis^x^ antigen gradually decreased over time. This prompted us to examine the *Fut4* and *Fut9* gene expression levels at different time points in MC38 cells. Interestingly, elevated mRNA levels of the *Fut4* gene (compared to MC38-MOCK cells) could be observed only within the first 48 h after isolation of the Lewis^x+^ cell population in MC38-FUT4 cells, followed by a dramatic decrease 96 h later (Supplementary Figure 2A). This could probably explain the subsequent decrease in cell-surface expression of Lewis^x^ 96 h after the initial enrichment (Supplementary Figure 2C). On the contrary, mRNA levels of the *Fut9* gene and the expression of Lewis^x^ in MC38-FUT9 cells remained stable over time, since we were able to detect exactly the same expression pattern even 30 days after sorting of the Lewis^x+^ cell population in these cells (Supplementary Figure 2B, C). Noticeably, the gradual decrease in Lewis^x^ neo-expression in MC38-FUT4 cells (with both a Lewis^x+^ and a Lewis^x-^ cell population being present at the same time) was not accompanied by a subsequent increase in the cell surface levels of other Lewis antigens (Supplementary Figure 2D), limiting the possibility of Lewis^x^ masking by other fucosyltransferases. Thus, loss of expression of the *Fut4* gene, in contrast to the *Fut9* gene, suggests that the expression of these specific fucosyltransferase genes may be differentially regulated in our cells. However, whether this loss of FUT4 is really due to unknown genetic or epigenetic factors influencing fucosyltransferase-specific gene expression levels in the context of colorectal cancer remains to be elucidated by future studies.

### The Lewis^x^ antigen is mainly carried by *N*-linked glycans and influences α2-3 sialylation in MC38-FUT4 and MC38-FUT9 cells

**Fig. 3.**
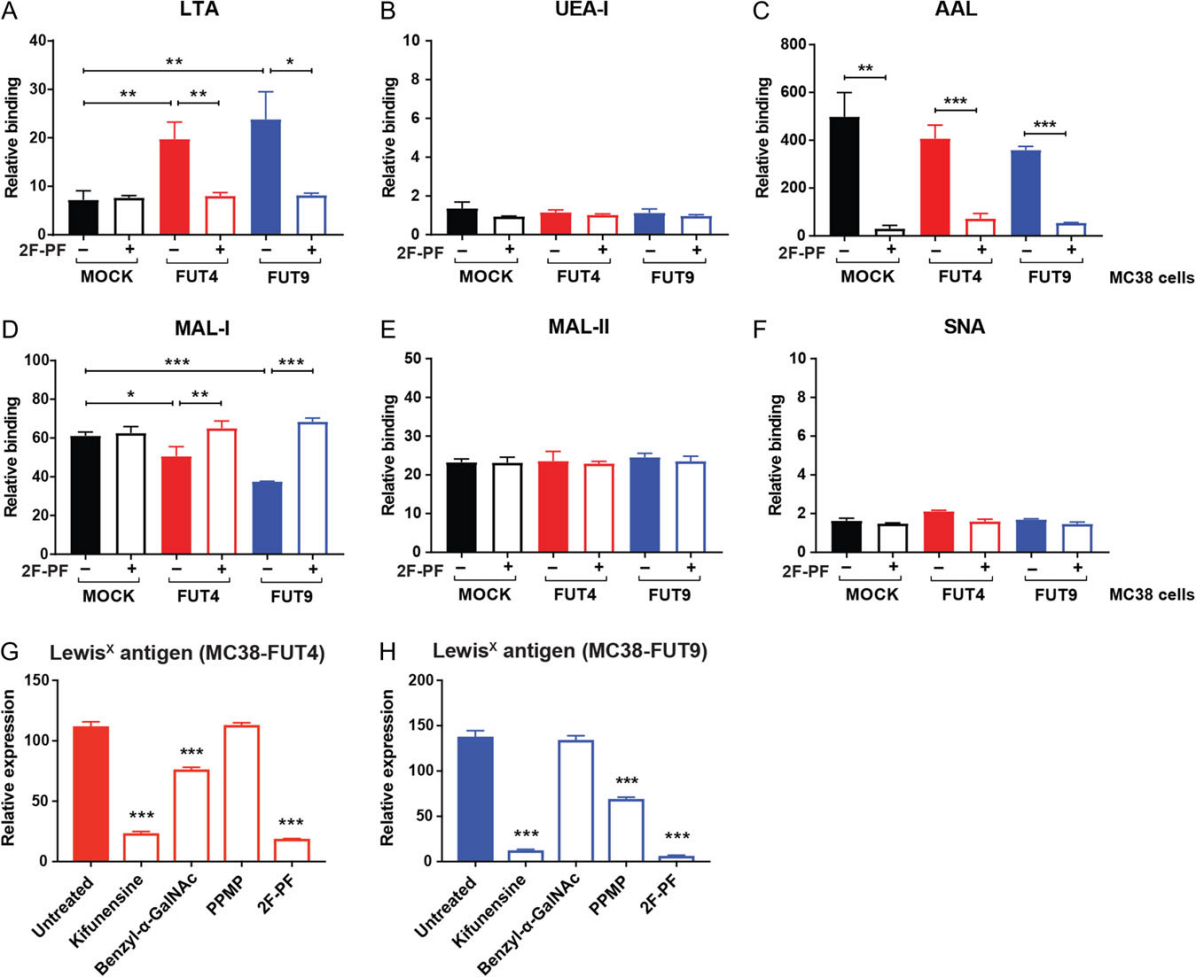
Lewis^x^ expressed on the surface of both MC38-FUT4 and MC38-FUT9 cells is mainly carried by *N*-linked glycans. **(A–F)** MC38-glycovariants, cultured for 48 h in the presence or absence of the fucosyltransferase inhibitor 2F-peracetyl fucose (2F-PF), were incubated for 30 min at 37°C with the indicated biotinylated plant lectins. The levels of antennary α1-3 fucosylation (LTA binding) (**A**), antennary α1-2 fucosylation (UEA-I binding) (**B**), core-fucosylation/ α1-3 fucosylation (AAL binding) (**C**), α2-3 sialylation on *N*-linked glycans (MAL-I binding) (**D**), α2-3 sialylation on *O*-linked glycans (MAA-II binding) (**E**) and α2-6 sialylation (SNA binding) (**F**) were examined by flow cytometry. **(G** and **H)** MC38-FUT4 (**G**) and MC38-FUT9 (**H**) cells were treated with different inhibitors of glycosylation for 48 h and changes in the cell-surface expression of Lewis^x^ were assessed by flow cytometry. Relative binding (**A–F**) or expression (**G, H**) represents the mean fluorescent intensities of three independent experiments after normalization to the binding of the corresponding secondary antibody alone. Statistical differences relative to the untreated condition are shown in **G** and **H**. Statistical significance was determined by a Student’s unpaired *t* test with Welch’s correction (*P < 0.05, **P < 0.01, ***P < 0.001).

After the generation and characterization of MC38 glyco-engineered cell lines, we wondered whether induction of the *Fut4* and *Fut9* genes, would affect the global cell surface glycosylation patterns of MC38 cells. For this purpose, we exploited a number of commonly used fucose- and sialic acid-recognizing plant lectins and assessed their binding to our MC38 glycovariants using flow cytometry (Figure 3A–F). As expected, we observed significantly higher binding of LTA (*Lotus Tetragonolobus* Agglutinin with specificity for terminal α1-3 fucosylation) to MC38-FUT4 and MC38-FUT9 cells compared to MC38-MOCK cells, which was completely reversed upon treatment of the cells with 2F-PF, confirming the presence of Lewis^x^ on these cells. However, no statistically significant differences in UEA-I (*Ulex Europaeus* Agglutinin-I recognizing terminal α1-2 fucosylation) or AAL binding (*Aleuria Aurantia* Lectin with specificity for core fucosylation and terminal α1-3 fucosylation) could be discerned among the examined MC38 cell lines, although a trend of decreased AAL binding to MC38-FUT4 and MC38-FUT9 cells was noted in the absence of 2F-PF treatment. At this point, due to the overlapping specificity of AAL for core fucosylation and terminal α1-3 fucosylation, we could not draw any conclusions from the AAL binding experiments. To our surprise, MC38-FUT4 and MC38-FUT9 cells displayed decreased binding of MAL-I (*Maackia Amurensis* Lectin-I recognizing α2-3 sialylation on *N*-glycans), while MAL-II (*Maackia Amurensis* Lectin-II with specificity for α2-3 sialylation on *O*-glycans) and SNA (*Sambucus Nigra* Agglutinin with specificity for α2-6 sialylation) displayed equal binding to all glycovariants. In addition, MAL-I binding to MC38-FUT4 and MC38-FUT9 cells could be rescued upon treatment of the cells with 2F-PF.

Based on these results, we hypothesized that the Lewis^x^ trisaccharide might be carried by *N*-linked glycan structures on the surface of MC38-FUT4 and MC38-FUT9 cells, thereby competing with α2-3 sialylation on *N*-glycans in these cells. Therefore, we treated MC38 glycovariants with different inhibitors of glycosylation and we examined changes in the surface expression of Lewis^x^ (Figure 3G, H). Interestingly, Lewis^x^ expression in both MC38-FUT4 and MC38-FUT9 cells was almost completely abrogated upon treatment with Kifunensine (*N*-glycosylation inhibitor), providing strong evidence that the Lewis^x^ epitope is predominantly carried by *N*-linked glycans. Of note, the decrease in Lewis^x^ expression due to Kifunensine treatment was comparable to the one observed after treatment of MC38 glycovariants with the general fucosyltransferase inhibitor, 2F-PF. Nevertheless, treatment of MC38 glycovariant cells with either benzyl-α-GalNAc (*O*-glycosylation inhibitor) or PPMP (glucosphingolipid synthesis inhibitor) revealed unique differences in the substrate specificity of the FUT4 and FUT9 enzymes, as Lewis^x^ appeared to be present, although to a lesser extent, also on *O*-glycans and glycosphingolipids in MC38-FUT4 and MC38-FUT9 cells, respectively.

### *N*-glycoprofiling of MC38 engineered cells reveals potent changes in their *N*-glycome composition

**Fig. 4.**
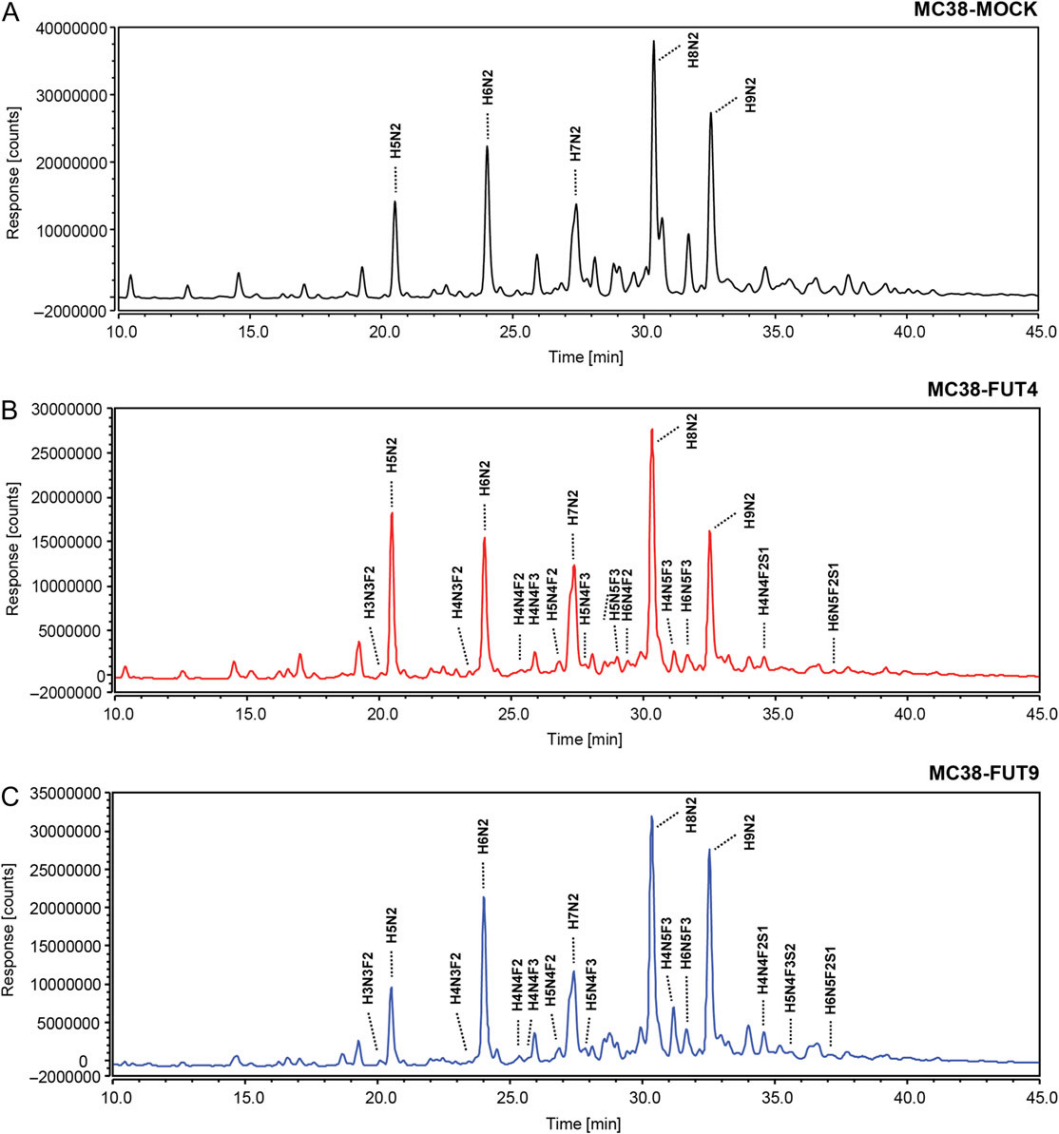
*N*-glycoprofiling of MC38-glycovariants using HILIC-(U)HPLC-FLR-ESI-MS. FLR chromatograms (10–45 min) corresponding to MC38-MOCK (**A**), MC38-FUT4 (**B**) and MC38-FUT9 (**C**) cells. Representative examples of di- or tri-fucosylated glycans bearing the Lewis^x^ antigen are depicted. The most abundant peaks of the FLR chromatograms represent the high-mannose *N*-glycans (H5N2/Man5, H6N2/Man6, H7N2/Man7, H8N2/Man8, H9N2/Man9) identified through our analysis. H = hexose (Hex); N = *N*-acetylhexosamine (HexNAc); F = fucose (Fuc); S = sialic/neuraminic acid (NeuAc).

**Fig. 5.**
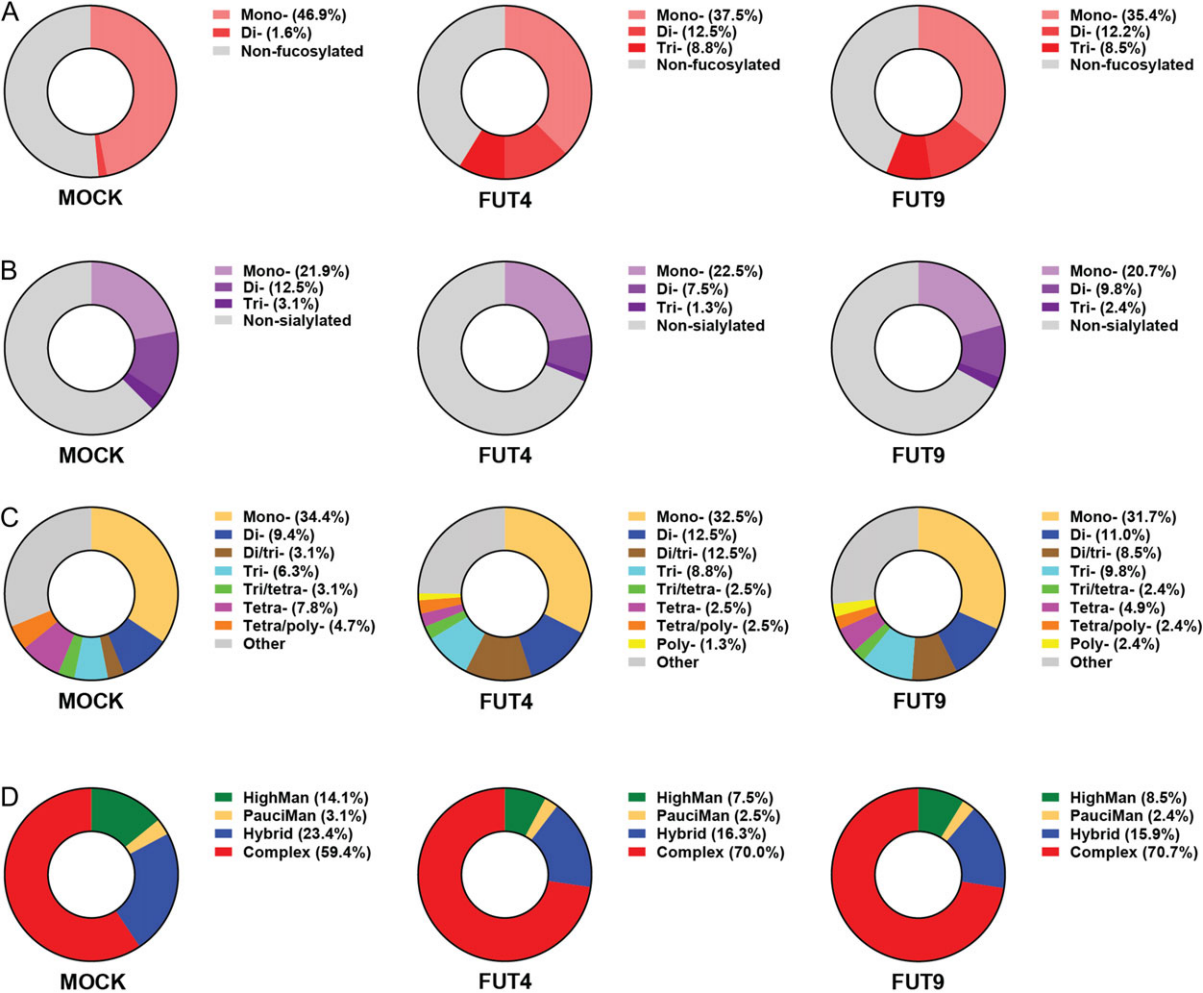
Relative abundance of distinct *N*-linked glycans in MC38-glycovariants identified by HILIC-(U)HPLC-FLR-ESI-MS. The possible *N*-glycan compositions were classified based on the specific traits examined, such as fucosylation (**A**), sialylation (**B**), antennarity (**C**) and glycan subtype (**D**), and the relative abundance was calculated for each cell line as indicated in Table I. “Other” represents the *N*-linked glycan compositions that could not be assigned according to the formulas provided in Table I.

Given that the Lewis^x^ antigen is mainly carried by *N*-linked glycans in MC38-FUT4 and MC38-FUT9 cells, we proceeded to detailed mass spectrometric *N*-glycoprofiling aimed at the identification of FUT4-, FUT9- or in general, Lewis^x^-specific alterations in the *N*-glycan composition of these cells. *N*-glycans released from whole MC38 cells with the use of PNGase F were labeled with procainamide and subsequently analyzed by HILIC-(U)HPLC-FLR-ESI-MS. All our MC38 glycosylation variants expressed core-fucosylated (fucose residue added in an α1-6 linkage to the innermost GlcNAc of the oligosaccharide core) *N*-glycans, as indicated by the AAL binding (Figure 3C). Nevertheless, the *N*-glycoprofiling analysis revealed that distinct di- or tri-fucosylated *N*-linked glycans bearing both core-fucose and the Lewis^x^ antigen (fucose residue added in an α1-3 linkage to distal GlcNAc residues of the oligosaccharide chain) were detected in MC38-FUT4 and MC38-FUT9 cells only, but not in MC38-MOCK cells (Figure 4A, B, C), validating our previous characterization of MC38-glycovariants using either the anti-Lewis^x^ specific antibody or the LTA plant lectin. Furthermore, we assessed the relative abundance of the identified *N*-linked glycans (Supplementary Table II) for each cell line separately, focusing on specific traits related to *N*-glycosylation (Table I), and classified the possible glycan compositions according to the paper by Holst et al. (2016). In agreement with our earlier observations, MC38-FUT4 and MC38-FUT9 cells displayed a higher relative abundance of di- or tri-fucosylated *N*-linked glycans compared to MC38-MOCK cells (Figure 5A), highlighting once again the presence of Lewis^x^ in these cells (representative example of the MS/MS analysis of MC38-FUT9 cells is provided as Supplementary Table III). Also, a decrease in the relative abundance of mono-fucosylated glycans (corresponding to core-fucosylation) was observed in MC38-FUT4 and MC38-FUT9 cells compared to MC38-MOCK cells, providing an explanation for the reduced AAL binding obtained before. Additionally, the decreased abundance of sialylated glycans (especially the di- and tri-sialylated ones) in MC38-FUT4 and MC38-FUT9 cells (Figure 5B) was consistent with the reduced MAL-I binding to these cells in comparison to MC38-MOCK cells. Therefore, these results imply that the biosynthesis and increased expression of Lewis^x^ have a negative impact both on core-fucosylation and on the *N*-linked sialic acids.

**Table I.** Formulas used for calculation of the relative abundance of specific traits defining the *N*-glycome of MC38-glycovariants, including fucosylation, sialylation, antennarity and glycan subtype

Examined traits	Calculation
Fucosylation	
Mono-	∑(Fuc = 1)
Di-	∑(Fuc = 2)
Tri-	∑(Fuc = 3)
Sialylation	
Mono-	∑(NeuAc = 1)
Di-	∑(NeuAc = 2)
Tri-	∑(NeuAc = 3)
Antennarity	
Mono-	∑(HexNAc ≤ 3)
Di-	∑(Hex = 5 and HexNAc = 4)
Di/Tri-	∑(Hex ≤ 5 and HexNAc = 5)
Tri-	∑(Hex = 6 and HexNAc = 5)
Tri/tetra-	∑(Hex ≤ 6 and HexNAc = 6)
Tetra-	∑(Hex = 7 and HexNAc = 6)
Tetra/poly-	∑(Hex ≤ 7 and HexNAc = 7)
Poly-	∑(Hex > 7 and HexNAc > 6)
Glycan subtype	
High-mannose (HighMan)	∑(Hex4-10HexNAc2)
Pauci-mannosidic (PauciMan)	∑(Hex1-3HexNAc2)
Hybrid	∑(Hex-HexNAc > 2)
Complex	∑(Hex-HexNAc ≤ 1)

The number of *N*-glycans matching the provided formulas was transformed into a relative abundance by dividing it to the total number of *N*-glycans identified for each cell line and multiplying it with 100%. Hex = hexose, H; HexNAc = *N*-acetylhexosamine, N; Fuc = fucose, F; NeuAc = sialic/neuraminic acid, S.

Next, we explored potential differences among the MC38-glycovariants in terms of *N*-glycan antennarity and specific glycan subtypes. Interestingly, compared to MC38-MOCK cells, MC38-FUT4 and MC38-FUT9 cells were characterized by a higher relative abundance of di- and tri-antennary *N*-linked glycans and by lower relative abundance of tetra- or tetra-/poly- *N*-linked glycans (Figure 5C). In addition, a small percentage of pure poly-antennary *N*-linked glycans could be identified only in MC38-FUT4 and MC38-FUT9 cells and not in the MC38-MOCK cell line. Furthermore, a switch in the balance of the known *N*-glycan types among the examined MC38 cells could be discerned, with MC38-FUT4 and MC38-FUT9 cells exhibiting a higher relative abundance of complex and a lower relative abundance of high-mannose or hybrid *N*-glycans compared to MC38-MOCK cells (Figure 5D). Collectively, these findings indicate that the emergence of Lewis^x^ on MC38-FUT4 and MC38-FUT9 cells induces potent changes in the cancer cell *N*-glycome, influencing antennarity and the respective glycan subtypes.

Finally, we were able to pinpoint a number of FUT4 and FUT9-specific *N*-glycan compositions through our analysis (Table II). For example, the composition H7N6F3 appeared only in the MC38-FUT9 cells and not in the MC38-MOCK or MC38-FUT4, while the composition H5N4F3S1 seemed to be exclusively expressed in the MC38-FUT4 cells. Together our data indicate that FUT4 and FUT9 may have overlapping, yet also unique biosynthetic properties regarding the modification of the substrate *N*-glycome in tumor cells.

**Table II.** List of possible *N*-linked glycans differing among the examined MC38-glycovariants.

Possible composition	MC38-MOCK	MC38-FUT4	MC38-FUT9
Neutral			
H3N6	−	+	+
** H4N8**	−	−	**+**
H6N3	+	+	−
H6N4	+	−	+
** H6N5**	−	−	**+**
** H6N7**	**+**	−	−
H7N3	+	+	−
** H7N4**	**+**	−	−
** H7N5**	**+**	−	−
** H7N8**	−	**+**	−
** H8N7**	−	−	**+**
** H9N3**	−	−	**+**
** H9N8**	−	**+**	−
H10N2	+	−	+
Fucosylated			
** H3N9F1**	−	**+**	−
H5N2F1	−	+	+
H5N3F1	−	+	+
** H5N5F1**	−	**+**	−
H7N6F1	+	+	−
** H9N5F1**	**+**	−	−
H3N3F2	−	+	+
H4N3F2	−	+	+
H4N4F2	−	+	+
H4N5F2	−	+	+
** H4N6F2**	**+**	−	−
H5N4F2	−	+	+
H4N4F3	−	+	+
H4N5F3	−	+	+
H5N4F3	−	+	+
** H5N5F3**	−	**+**	−
** H7N6F3**	−	−	**+**
Sialylated			
** H3N4S1**	**+**	−	−
H4N3S1	−	+	+
H4N4S1	−	+	+
H4N5S1	−	+	+
** H7N6S1**	**+**	−	−
** H4N4S2**	**+**	−	−
H4N5S2	−	+	+
** H5N5S2**	−	**+**	−
H7N6S2	+	−	+
** H5N5S3**	−	−	**+**
Mixed			
** H4N3F1S1**	**+**	−	−
H4N4F2S1	−	+	+
H5N4F2S1	−	+	+
** H5N4F3S1**	−	**+**	−
** H5N5F1S1**	−	**+**	−
H7N6F1S1	+	−	+
** H8N5F1S1**	**+**	−	−
H5N4F1S2	+	+	−
** H5N4F3S2**	−	−	**+**
** H6N5F1S2**	**+**	−	−
H7N5F1S2	+	−	+
** H7N7F1S2**	−	−	+
** H6N5F1S3**	**+**	−	−
** H8N7F1S4**	−	−	**+**

*N*-glycans unique to only one of our glycovariant cell lines are highlighted in bold; (+) indicates presence, (–) indicates absence. Hex = hexose, H; HexNAc = *N*-acetylhexosamine, N; Fuc = fucose, F; NeuAc = sialic/neuraminic acid, S.

### Lewis^x^ antigen neo-expression is functionally relevant and can be recognized by designated lectin receptors

**Fig. 6.**
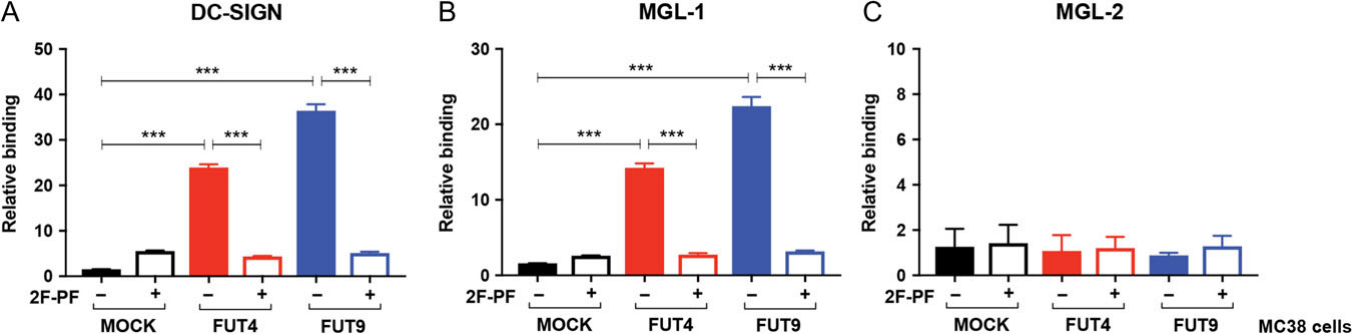
Binding of C-type lectin receptors to MC38-glycovariants. MC38 cells, cultured for 48 h in the presence or absence of the fucosyltransferase inhibitor 2F-peracetyl fucose (2F-PF), were incubated for 30 min at 37°C with human DC-SIGN-Fc (DC-SIGN) (**A**), mouse MGL-1-Fc (MGL-1) (**B**) or mouse MGL-2-Fc (MGL-2) (**C**) and lectin binding was assessed by flow cytometric analysis. Relative binding represents the mean fluorescent intensities of three independent experiments after normalization to the binding of the secondary antibody alone. Statistical significance was determined by a Student’s unpaired *t* test with Welch’s correction (***P < 0.001).

After our thorough examination of the *N*-glycome of the MC38-FUT4 and MC38-FUT9 glycovariant cell lines, we next determined the functional relevance of cell surface Lewis^x^ and evaluated the binding of specific C-type lectin receptors (CLRs) to our MC38 glycovariants in vitro. For this purpose, we used human DC-SIGN (hDC-SIGN) (van Gisbergen et al. 2005) and mouse MGL-1 (mMGL-1) (Montero-Barrera et al. 2015), as these CLRs are well-known Lewis^x^-recognizing receptors that are physiologically expressed on antigen-presenting cells, such as dendritic cells and macrophages. As a negative control, we chose mouse MGL-2 (mMGL-2) (Singh et al. 2009), taking advantage of its lack of specificity for the Lewis^x^ determinant. Compared to the MC38-MOCK cells, a significant increase in the binding of hDC-SIGN (Figure 6A) and mMGL-1 (Figure 6B), but not mMGL-2 (Figure 6C), could be observed to MC38-FUT4 and MC38-FUT9 cells. Since there was no CLR binding to MC38-MOCK cells lacking Lewis^x^ and the binding was completely lost upon treatment of MC38-FUT4 and MC38-FUT9 cells with 2F-PF, we concluded that the receptor–ligand interactions were CLR-specific and Lewis^x^-mediated.

Taken together, our results signify that the Lewis^x^ determinant synthesized by the FUT4 and FUT9 enzymes following CRISPR-dCas9-VPR-induced gene activation is expressed on the cell-surface in a physiological manner, thus maintaining the full range of its functional and biologically relevant properties.

## Discussion

Since the initial discovery of clustered regularly interspaced short palindromic repeats (CRISPR) as the adaptive immune system of bacteria and archaea (Wiedenheft et al. 2012), the CRISPR-Cas9 system has been rapidly transformed to a powerful genome-engineering tool with various research applications, including gene editing (gene knock out/in) and gene expression programming (transcriptional gene activation/suppression) among others. The greatest advantage of this system is that the Cas9 nuclease (wild type or deactivated) can be directed to virtually everywhere in the genome with the use of short guide RNAs which, based on DNA sequence complementarity, specifically target the genomic region of interest (Hsu et al. 2014).

Undoubtedly, research investigations focused on protein and lipid glycosylation can be vastly facilitated by the availability of the CRISPR-Cas9 technology. Besides the direct glycan analysis techniques that have been developed and mainly used so far, the glycosylation machinery within the cells and various functional properties of the glycome can be now further explored through the genetic dissection of distinct glycan biosynthetic pathways. For example, a GlycoCRISPR resource became recently available (Narimatsu et al. 2018), including a validated gRNA library for CRISPR-Cas9 targeting of the human glycosyltransferase genome and enabling potential gene knock-out applications suitable for 167 human enzymes involved in glycan biosynthesis.

In the present study, we exploited the CRISPR-dCas9-VPR system for transcriptional activation of certain α1-3 fucosyltransferase genes, *Fut4* (Giordano et al. 2015) and *Fut9* (Auslander et al. 2017), playing a major role in colorectal cancer pathogenesis. According to our findings, transcriptional activation of the *Fut4* and *Fut9* genes in MC38 cells resulted in specific neo-expression of the Lewis^x^ antigen on the cell surface. With this, we concluded that the CRISPR-dCas9-VPR-mediated transcriptional gene activation of FUT4 and FUT9 in MC38 cells can be reliably linked to the tumor-associated fucosyltransferase function, with Lewis^x^ emerging as the main fucosylated epitope that these enzymes synthesize in common in the context of colorectal cancer.

Regarding the substrate specificity, it is known that FUT9 preferentially fucosylates the GlcNAc residue at the distal lactosamine unit of the polylactosamine chain, whereas FUT4 transfers a fucose to the GlcNAc residue at the inner lactosamine unit (Nishihara et al. 1999). However, the preferred carrier molecules of Lewis^x^, synthesized by FUT4 and FUT9 in colorectal cancer cells, have been scarcely studied so far. In our MC38 model system, Lewis^x^ was predominantly carried by *N*-linked glycans in both MC38-FUT4 and MC38-FUT9 cells. Nevertheless, critical differences in the substrate specificity of the FUT4 and the FUT9 enzymes were also detected in our analysis, since *O*-glycans and glycosphingolipids appeared to be the secondary preferential glycan carriers of Lewis^x^ in MC38-FUT4 and MC38-FUT9 cells, respectively (Nishihara et al. 2003, Sperandio et al. 2009).

Defining the cancer-specific biosynthetic properties of FUT4 and FUT9 is fundamental for the interpretation of the biological functions that these enzymes exert within a tumor cell. Strikingly, *N*- and *O*-glycans, as well as glycosphingolipids, have been associated with different functional properties during malignant cell transformation or metastasis (Oliveira-Ferrer et al. 2017). For example, *N*-glycans regulate the protein conformation as well as the intracellular signaling properties of tumor-associated growth factor receptors, such as the epidermal growth factor receptor (EGFR) and the transforming growth factor β (TGF-β) receptor (Takahashi et al. 2016). Therefore, *N*-glycans have been recently proposed as therapeutic targets in colorectal cancer (de Freitas and Morgado-Diaz 2016). *O*-glycans have specialized functions in cell adhesion and lipid metabolism (Chia et al. 2016), whereas glycosphingolipids play a major role in anti-cancer drug resistance (Gouaze-Andersson and Cabot 2006) and have been implicated in the maintenance of cancer stemness (Liang et al. 2017).

More specifically, we demonstrate here that de novo synthesis of the fucosylated Lewis^x^ antigen by FUT4 and FUT9 leads to potent alterations in the *N*-glycome of colorectal cancer cells, with a negative impact exerted on core-fucosylation and the α2-3 sialylation levels. This observation highlights existing monosaccharide-, enzyme- and site-specific antagonistic effects occurring during the early stages of *N*-glycan biosynthesis, the dynamics of which are commonly missed or undermined due to the lack of techniques that are sensitive enough for detection of real-time changes in the cancer cell glycome. In more detail, changes in the *N*-glycan composition of cancer cells have been shown to affect the subsequent interaction with immune cells (Nardy et al. 2016). For instance, hypersialylated tumor cells can engage different Siglec (sialic acid-binding immunoglobulin-type lectins) receptors expressed on myelomonocytic cells and inhibit successful immunosurveillance, depending on the stage of tumor growth and the surrounding microenvironment (Laubli et al. 2014). However, it is still unknown whether increased expression of FUT4 and FUT9, influencing the abundance of α2-3-linked sialic acids on *N*-glycans, is associated with decreased Siglec binding to colorectal cancer cells and modulation of the anti-tumor immune response in vivo. Conversely, the FUT4- and FUT9-mediated synthesis of DC-SIGN and MGL-1 ligands containing the Lewis^x^ epitope hints to potential implication of these enzymes in the induction of an immunosuppressive tumor microenvironment in colorectal cancer. This is supported by the fact that DC-SIGN binding to Lewis antigens overexpressed by cancer cells, results in induction of T helper 2 (Th2) and regulatory T (Treg) cells, decreased activity of natural killer (NK) cells and increased production of anti-inflammatory cytokines, such as interleukin-10 (IL-10) (Rodríguez et al. 2018). Although the exact involvement of MGL-1 in the tumor growth remains elusive, expression of MGL-1 by macrophages has been associated with increased production of IL-10 and acquisition of an anti-inflammatory role in a mouse model of experimental colitis (Saba et al. 2009).

Additionally, the induction of complex *N*-glycan synthesis in MC38 cells upon FUT4 and FUT9 induction raises a possible role for these fucosyltransferases in the metabolic control of colorectal cancer cells. So far, the contribution of complex *N*-glycans to the altered metabolic status of cancer cells, especially through sensitization to different growth factors, has been merely linked to the expression of *Mgat5* (Golgi beta1,6*N*-acetylglucosaminyltransferase V) (Mendelsohn et al. 2007), although more glycosyltransferases might be directly or indirectly involved in this process (Munkley and Elliott 2016). Moreover, LacNAc stretches on complex *N*-glycans are major ligands for Galectins involved in tumorigenesis, such as Galectin-1, -3 and -8 (Patnaik et al. 2006). Nevertheless, whether the observed changes in *N*-glycan antennarity and abundance of complex *N*-glycans in MC38-FUT4 and MC38-FUT9 cells influence the metabolic or Galectin binding properties (Chou et al. 2018) of colorectal cancer cells, requires further investigation.

In summary, we here show that application of the CRISPR-dCas9-VPR technology to augment glycosyltransferase expression in primary cells or cell lines can provide a better insight into potential alterations occurring during malignant cell transformation, and thus, unravel multiple aspects related to the structural or functional properties of the mammalian glycome in health and disease.

## Materials and methods

### pX330-Delta_Puro cloning

The parental pX330 sgRNA Cas9 expression vector from the Zhang lab was obtained from Addgene (ID42230, Addgene, Cambridge, USA). The Puromycin resistance cassette from pX260 (Zhang lab, ID42229, Addgene) was PCR amplified with Phusion High Fidelity polymerase (New England BioLabs, Massachusetts, USA) using forward primer: 5′GGTCGCCCGACGCCCTTACGTCCAGCCAAGCTTAG3′, and reverse primer: 5′TCACTGAGGCCGCCCCGTACTATGGTTGCTTTGAC3′ (Sigma Aldrich, Missouri, USA). The PCR product encoding the Puromycin marker was cloned into the pX330 vector after backbone linearization using two neighboring *Sma*I restriction sites (New England BioLabs) and InFusion cloning (Clontech, California, USA). Next, the Cas9 coding sequence was removed from the vector backbone by *Nco*I and *Eco*RI (New England BioLabs) digests. A linker consisting of hybridized oligonucleotides was cloned into the *Nco*I and *Eco*RI sites to circularize the vector using InFusion cloning. Oligonucleotide sequences used were, sense: 5′ATCGCTATTACCATGGAACAATGAGTCTGCATCAAG3′, anti-sense: 5′CGAGCTCTAGGAATTCTTGATGCAGACTCATTGTTC3′ (Sigma Aldrich).

### gRNA design and cloning

As previously described (Chavez et al. 2015), the principle behind Cas9-mediated transcriptional programming is that one or multiple gRNA sequences specifically target the promoter region of the gene of interest in close proximity to the transcriptional start site, resulting in direct recruitment of the catalytically inactive dCas9 nuclease to this site. The subsequent interaction between the VPR (VP64-p65-Rta) tripartite activation domain and RNA polymerase II and/or other transcription factors drives the induction of expression of the (glyco)gene of interest (Figure 1A). For this reason, the Eukaryotic Promoter Database (EPD) (https://epd.vital-it.ch/index.php) was used for selecting the promoter regions of the murine *Fut4* and *Fut9* genes. 20-nucleotide gRNAs sequences complementary to the promoter of each target fucosyltransferase gene were designed and validated with the E-CRISP (http://www.e-crisp.org/E-CRISP/) and the CRISPR design prediction tools (http://crispr.mit.edu/), respectively (Supplementary Table I). The Basic Local Alignment Search Tool (BLAST) (https://blast.ncbi.nlm.nih.gov/Blast.cgi) and the transcription factor binding-site prediction tool (Tfsitescan) (http://www.ifti.org/cgi-bin/ifti/Tfsitescan.pl) were exploited for quality control of the selected gRNAs and to avoid interference with major transcription factor-binding sites (Figure 1B). Selected gRNAs (Supplementary Table I) were ordered (Invitrogen, California, USA) and cloned afterwards into the pX330-Delta_Puro expression vector, as described by Ran et al. (2013). Finally, the SP-dCas9-VPR plasmid from the Church lab (ID63798, Addgene) was used for expression of the dCas9-VPR protein.

### Cell culture and transfections

Wild type MC38 cells (murine colon carcinoma), Panc02 (mouse pancreatic ductal adenocarcinoma) (gift from Prof. Dr. M. van Egmond, Amsterdam UMC, Vrije Universiteit Amsterdam, Dept. of Surgery and Molecular Cell Biology and Immunology, Cancer Center Amsterdam), LL/2 (Lewis lung carcinoma) (gift from Prof. Dr. T. Freire, UdelaR, Montevideo, Uruguay) and glyco-engineered cell lines were maintained in high glucose Dulbecco’s Modified Eagle’s Medium (Gibco, Life Technologies, California, USA) supplemented with 10% FBS premium (Biowest, Nuaillé France) and penicillin/streptomycin (Lonza, Bazel, Switzerland). Cells were maintained at 37°C and 5% CO_2_ in a humidified incubator and tested for mycoplasma infection monthly. Cells were transfected in 24-well plates seeded with 50.000 cells per well. 400 ng of the SP-dCas9-VPR-expressing plasmid plus 80 ng of the empty (MC38-MOCK cells) or 80 ng of the FUT4/FUT9 gRNA-expressing PX330-Puro_Delta plasmid (MC38-FUT4/FUT9 cells) were delivered simultaneously to the corresponding wells (protocol adapted from Chavez et al.) together with Lipofectamine LTX (Invitrogen), according to manufacturer’s instructions. During transient transfections, cells were grown for 48 h before being assayed by flow cytometry. Stable cell lines were selected with 6 μg/mL Puromycin (Sigma Aldrich) for expression of the corresponding gRNA construct, which was applied to the cells 48 h post-transfection.

### Enrichment of Lewis^x+^ MC38 cells

Manual cell separation (MACS) with the use of anti-CD15 (Lewis^x^) microbeads (Miltenyi Biotec, San Diego, USA) was performed according to manufacturer’s instructions to enrich for the Lewis^x+^ MC38-FUT4 and MC38-FUT9 glyco-engineered cells 10 days after initial transfection. From this point and due to deviation in the stability of the *Fut4* gene expression levels, enrichment of the Lewis^x+^ fraction of MC38-FUT4 cells specifically was conducted every 2–3 days in order to maintain Lewis^x^ neo-expression in the MC38-FUT4 until the completion of our study. MC38-FUT9 were stable throughout our analysis and did not require any further enrichment steps.

### qRT-PCR analysis

MC38, Panc02 and LL/2 cell-lysis and mRNA isolation was performed with the mRNA capture kit (Roche, Bazel, Switzerland) and cDNA synthesis was performed using the Reverse Transcription System Kit (Promega, Wisconsin, USA), according to manufacturer’s instructions. Synthesized cDNA samples together with the KAPA SYBR® FAST Universal 2X qPCR Master Mix and specific qPCR primers were utilized for each qRT-PCR reaction. Precisely, qPCR primer sequences used for cDNA amplification were as follows: GAPDH forward primer: CCTGCACCACCAACTGCTTAG; GAPDH reverse primer: CATGGACTGTGGTCATGAGCC; FUT4 forward primer: CAGCCTGCGCTTCAACATC; FUT4 reverse primer: CGCCTTATCCGTGCGTTCT; FUT9 forward primer: ATCCAAGTGCCTTATGGCTTCT; FUT9 reverse primer: TGCTCAGGGTTCCAGTTACTCA. qRT-PCR for individual genes was ran and analyzed on the CFX96 Real-Time PCR Detection System (BIORAD, California, USA), with all target gene expression levels normalized to GAPDH (*M. Musculus*) mRNA levels.

### Flow cytometry

The primary antibodies used were: anti-Lewis^a^ (clone T174, Calbiochem, San Diego, USA), anti-Lewis^b^ (clone T218, Calbiochem), anti-Lewis^x^ (clone P12, Calbiochem), anti-Lewis^y^ (clone F3, Abcam, Cambridge, United Kingdom), anti-sialyl-Lewis^x^ (clone CSLEX, BD Pharmingen, San Jose, USA) and anti-CD65s (clone VIM-2, LabNed, the Netherlands). The biotinylated lectins used were: LTA (*Lotus Tetragonolobus* Agglutinin α-1,3-linked fucose), UEA-I (*Ulex Europaeus* Agglutinin-I, α-1,2-linked fucose), AAL (*Aleuria Aurantia* Lectin, α-1,6-linked and α-1,3-linked fucose), MAL-I (*Maackia Amurensis* Lectin-I, α-2,3-linked sialic acid residues on *N*-glycans), MAL-II (*Maackia Amurensis* Lectin-II, α-2,3-linked sialic acid residues on *O*-glycans) and SNA (*Sambucus Nigra* Agglutinin, α-2,6-linked sialic acid residues), all ordered from Vector Labs (Burlingame, USA). The plant lectin specificity provided above was confirmed by the Functional Glycomics Database (CFG): http://www.functionalglycomics.org/. The C-type lectin-Fc constructs were generated as previously described (Appelmelk et al. 2003) (Singh et al. 2009).

Cells were harvested, washed and resuspended in phosphate buffered saline (PBS) or Hank’s balanced salt solution (HBSS) (Gibco, Life Technologies) containing 0.5% bovine serum albumin (BSA) at 1 × 10^6^ cells/mL. 50.000 cells were added to each well of a 96-well V-bottom plate and incubated with 5 μg/mL of the respective primary antibodies, biotinylated lectins or 10 μg/mL of the C-type lectin-Fc constructs for 30 min at 4°C or 37°C, respectively. Cells were washed and resuspended in 50 μL PBS or HBSS (0.5% BSA) containing the corresponding secondary reagents and incubated for 30 min at 37°C. Goat anti-mouse IgG FITC was used for anti-Lewis^a^ (Jackson Labs, California, USA) and goat anti-mouse IgM FITC (Jackson Labs, USA) was used for the other primary antibodies. Streptavidin-APC (Biolegend, California, USA) was used to detect plant lectin binding and goat anti-human IgG-Fc FITC (Jackson Labs) for the C-type lectin-Fc constructs. Cells were washed with 100 μL PBS or HBSS (0.5% BSA) and subsequently resuspended in 100 μL PBS or HBSS (0.5% BSA). Fluorescence intensities were measured using either the FACSCalibur^TM^ (BD Bioscience, New Jersey, USA) or the CyAn ADP (Beckman Coulter, Brea, USA) flow cytometers and cytometric data analysis was performed with the FlowJo V10 software.

### Inhibitors of glycosylation

MC38 cells were treated with 10 μg/mL Kifunensine (mannosidase I inhibitor) (Sigma Aldrich), 2 mM Benzyl-α-GalNAc (*O*-glycosylation inhibitor) (Sigma Aldrich), 1 μM 1-phenyl-2-palmitoylamino-3-morpholino-1-propanol (PPMP, glucosphingolipid synthesis inhibitor) (Enzo Life Sciences, Brussels, Belgium) or 0–100 μg/mL 2F-peracetyl fucose (2F-PF) (general fucosyltransferase inhibitor) (Merck, New Jersey, USA) according to manufacturer’s instructions for 48 h before cells were analyzed by flow cytometry.

### *N*-glycan release

MC38 cells were washed three times with PBS and dried cell pellets (3 × 10^6^/cell line) were frozen until further use. Upon thawing, the cell pellets were resuspended in 100 μL pure water and were homogenized for 45 min in a sonication bath in order to disrupt the cell membranes. After sonication, the samples were centrifuged (500 × *g*, 15 min) and 17.5 μL of pure water was added to each sample and mixed. 5 μL of reaction buffer (250 mM sodium phosphate buffer; pH 7.5) and 1.25 μL of denaturation buffer (2% SDS in 1 M β-mercaptoethanol) was added. The samples were incubated for 10 min at 100°C to denature the proteins. After cooling the samples to room temperature, 1.25 μL of Triton X-100 and 500 units of PNGase F (QABio, California, USA) were added to each sample. Samples were vortexed and incubated overnight at 37°C. The released glycans were then converted to aldoses with 0.1% formic acid, filtered through a protein-binding membrane and dried (Royle et al. 2006).

### Glycan labeling

Released *N*-glycans were fluorescently labeled by reductive amination with procainamide as described previously (Kozak et al. 2015) using LudgerTag^TM^ Procainamide Glycan Labelling Kit (LT-KPROC-24). Briefly, samples in 10 μL of pure water were incubated for 60 min at 65°C with procainamide labeling solution. The procainamide labeled samples were cleaned-up using a Ludger clean plate (LC-PROC-96). The purified procainamide labeled *N*-glycans were eluted with pure water (100 μL). The samples were dried by vacuum centrifugation and resuspended in pure water (100 μL) for further analysis.

### Liquid chromatography mass-spectrometry (LC–MS)

Procainamide labeled samples and system suitability standards were analyzed by HILIC-(U)HPLC-ESI-MS with fluorescence detection. Samples were injected in 24% pure water/76% acetonitrile (injection volume 25 μL) onto an ACQUITY UPLC® BEH-Glycan 1.7 μm, 2.1 × 150 mm column at 40°C on a Ultimate 3000 UHPLC instrument (Thermo Scientific, Massachusetts, USA) with a fluorescence detector (λex = 310 nm, λem = 370 nm). The running conditions used were: Solvent A was 50 mM ammonium formate pH 4.4 made from Ludger Stock Buffer (Ludger), and solvent B was acetonitrile. Gradient conditions were: 0–38.5 min, 76–58% B; 38.5–40.5 min, 58–40.5% B at a flow rate of 0.4 mL/min; 40.5–42.5 min, 40% B at a flow rate of 0.25 mL/min; 42.5–44.5 min, 40–76% B at a flow rate of 0.25 mL/min; 44.5–50.5 min, 76% B at a flow rate of 0.25 mL/min; 50.5–51.5 min, 76% B at a flow rate of 0.25 mL/min; 51.5–55 min, 76% B at a flow rate of 0.4 mL/min. The UHPLC system was coupled on-line to an AmaZon Speed ETD electrospray mass spectrometer (Bruker Daltonics, Bremen, Germany) with the following settings: source temperature 250°C, gas flow 10 L/min; Capillary voltage 4500 V; ICC target 200,000; maximum accumulation time 50 ms; rolling average 2; number of precursors ions selected 3, release after 0.2 min; Positive ion mode; Scan mode: enhanced resolution; mass range scanned, 180–1500; Target mass, 700. A glucose homopolymer ladder labeled with procainamide (Ludger) was used as a system suitability standard as well as an external calibration standard for GU allocation. ESI-MS and MS/MS data analysis was performed using Bruker Compass DataAnalysis V4.1 software and GlycoWorkbench software (Ceroni et al. 2008). The relative abundance of identified *N*-linked glycans was determined as follows: (Number of *N*-glycans matching the formulas listed in Table I/total number of *N*-glycans identified for each cell line) ∗ 100%.

### Statistical analysis

All statistical analyses were performed with the Prism software (GraphPad V8 Software). Statistical significance was determined by a Student’s unpaired *t* test with Welch’s correction: *P < 0.05, **P < 0.01, *** *P* < 0.001.

## Supplementary Material

Supplementary Data

## Abbreviations

2F-PF: 2F-peracetyl fucose
AAL: *Aleuria Aurantia* lectin
BLAST: basic local alignment search tool
BSA: bovine serum albumin
CLR: C-type lectin receptor
CRISPR: clustered regularly interspaced short palindromic repeats
DC-SIGN: dendritic cell-specific ICAM3-grabbing non-integrin
EGFR: epidermal growth factor receptor
EPD: eukaryotic promoter database
ESI: electrospray ionization
FBS: fetal bovine serum
FLR: fluorescence
*Fut4*: fucosyltransferase 4 gene
FUT4: fucosyltransferase 4 protein
*Fut9*: fucosyltransferase 9 gene
FUT9: fucosyltransferase 9 protein
GlcNAc: *N*-acetylglucosamine
gRNA: guide RNA
HBSS: Hank’s balanced salt solution
HILIC: hydrophilic interaction chromatography
HPLC: high performance liquid chromatography
IL-10: Interleukin-10
LTA: *Lotus Tetragonolobus* Agglutinin
MAL-I: *Maackia Amurensis* Lectin-I
MAL-II: *Maackia Amurensis* Lectin-II
*Mgat5*: golgi beta1,6*N*-acetylglucosaminyltransferase V gene
MGL-1: macrophage galactose-type lectin-1
MGL-2: macrophage galactose-type lectin-2
mRNA: messenger RNA
MS: mass spectrometry
NK: natural killer
PPMP: 1-phenyl-2-palmitoylamino-3-morpholino-1-propanol
Siglec: sialic acid-binding immunoglobulin-type lectin
SNA: *Sambucus Nigra* agglutinin
SSEA-1: stage-specific embryonic antigen-1
TGF-β: transforming growth factor-β
Th: T helper
Treg: T regulatory
UEA-I: *Ulex Europaeus* Agglutinin-I
VPR: VP64-p65-Rta
