# Supplementary material for: Transcriptional activation of fucosyltransferase (FUT) genes using the CRISPR-dCas9-VPR technology reveals potent N-glycome alterations in colorectal cancer cells
Source: Glycobiology. 2018 Oct 22;29(2):137–50. doi: 10.1093/glycob/cwy096 (PMC6330019; doi:10.1093/glycob/cwy096)
Supplement: Supplementary Data [file cwy096_supporting_information_blanas_et_al.pdf]

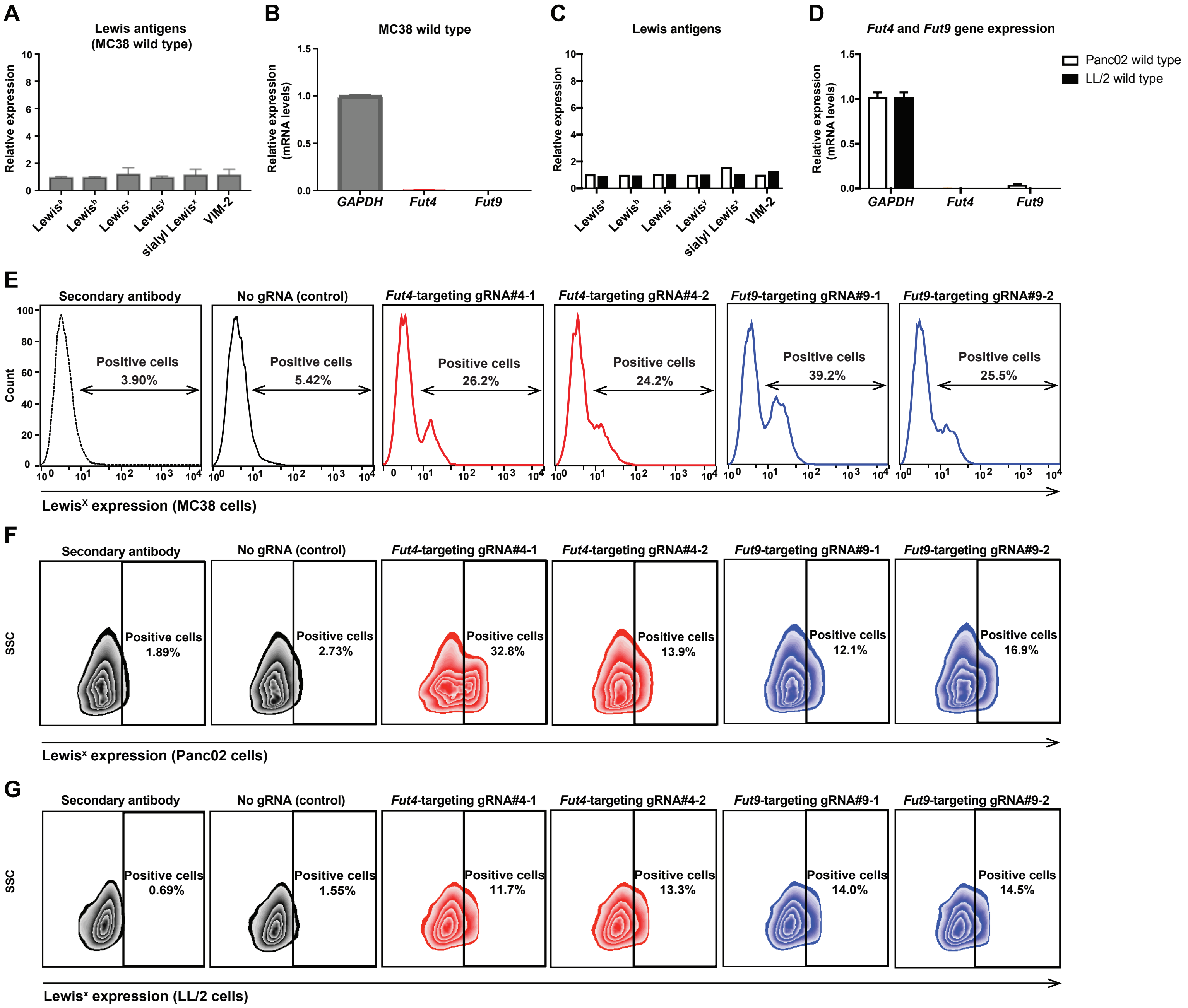

Supplementary Figure 1: Functionality of the designed *Fut4*- and *Fut9*-targeting gRNA sequences in different mouse cancer models. Expression of type I (Lewis^a, Lewis^b) and type II (Lewis^x, Lewis^y, sialyl Lewis^x and VIM-2) Lewis antigens on the surface of MC38 (**A**), Panc02 and LL/2 (**C**) wild type cells. Mean fluorescent intensities were normalized to the binding of the secondary antibody alone. Assessment of the relative mRNA levels of the *Fut4* and *Fut9* genes in MC38 (**B**), Panc02 and LL/2 (**D**) wild type cells. Expression was normalized to the housekeeping gene *GAPDH* (*M. musculus)*. Expression of Lewis^x on the surface of MC38 (**E**), Panc02 (**F**) and LL/2 (**G**) cells that were transiently (for 48 hours) transfected with Sp-dCas9-VPR and the selected *Fut4*- or *Fut9*-targeting gRNA sequences (listed in **Supplementary Table I**). As a control, cells were transfected with the Sp-dCas9-VPR-expressing plasmid alone (no gRNA).

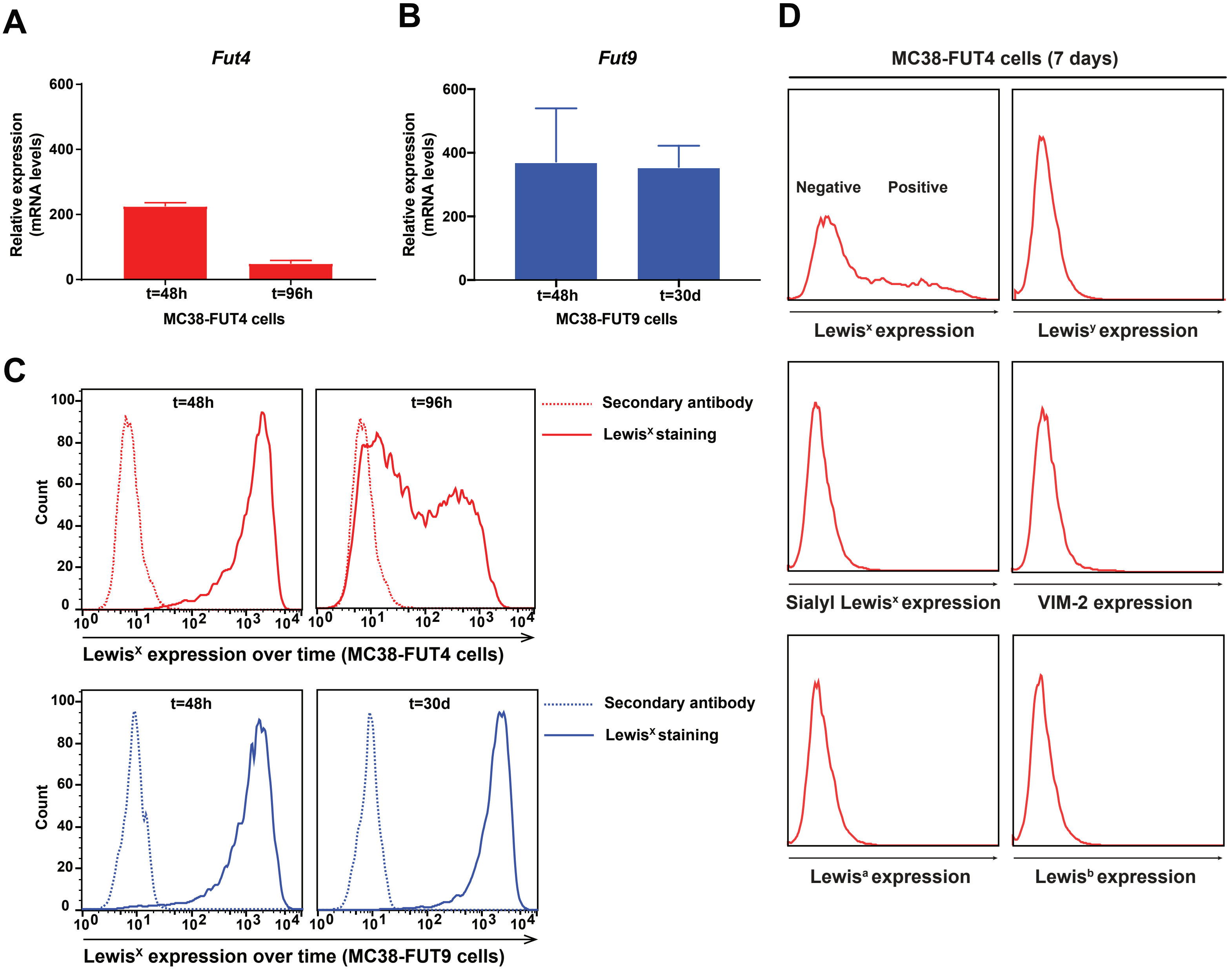

Supplementary Figure 2: Stability of the *Fut4* and *Fut9* transcriptional gene activation and Lewis^{x} neo-expression in MC38-glycovariants. **A**) Assessment of the *Fut4* gene expression (mRNA levels) in MC38-FUT4 cells 48 and 96 hours after enrichment of the Lewis^{x+} cell population using anti-CD15 magnetic microbeads. Statistical differences relative to MC38-MOCK cells are depicted. **B**) Assessment of the *Fut9* gene expression (mRNA levels) in MC38-FUT9 cells 48 hours and 30 days after isolation of the Lewis^{x+} cell population using anti-CD15 magnetic microbeads. Statistical differences relative to MC38-MOCK cells are depicted. **C**) Lewis^{x} expression on the surface of MC38-FUT4 and MC38-FUT9 cells at different time points (48, 96 hours or 30 days) after initial enrichment of the Lewis^{x+} cell population. **D**) Gradual loss of Lewis^{x} neo-expression in MC38-FUT4 cells is not accompanied by elevated levels of other relevant type I or type II Lewis antigens. The cell surface expression of Lewis antigens was examined in MC38-FUT4 cells one week after isolation of the Lewis^{x+} cell population using anti-CD15 magnetic microbeads, when both a Lewis^{x+} and a Lewis^{x-} cell fraction are still present.

Supplementary Table I: List of selected and validated gRNAs targeting sequences in close proximity to the transcriptional start site (TSS) of the murine *Fut4* and *Fut9* genes, respectively.

Target gene	gRNA	gRNA sequence	Length (nt)	Targeted chromosome	Targeted genomic location (bp)	Distance from TSS (bp)
Murine *Fut4*	gRNA#4-1	GAG GTA TCC AGT GCA AGG CG	20	9	14752290 - 14752309	-187
	gRNA#4-2	GCC AGC GGG GCG CTG TTT CT	20	9	14752204 - 14752223	-101
Murine *Fut9*	gRNA#9-1	GCA TAT CGG AGA CGC AGC AA	20	4	25800227 - 25800246	-1
	gRNA#9-2	GCC TCC CGA CTC AAC ACA CG	20	4	25800140 - 25800159	+86

Supplementary Table II: List of possible *N*-linked glycan compositions identified in MC38-glycovariants using HILIC-(U)HPLC-FLR-ESI-MS. Information about the corresponding registered or calculated mass [*m/z*]$^+$, charge [H$^+$] and average GU value is provided for all the glycan compositions. For each cell line, the possible *N*-linked glycans were categorized into four groups, including neutral (non-fucosylated/non-sialylated), fucosylated (mono-, di- or tri-fucosylated), sialylated (mono-, di- or tri-sialylated) and mixed (fucosylated and sialylated) compositions. Hex= Hexose, H; HexNAc= *N*-acetylhexosamine, N; Fuc= Fucose, F; NeuAc= Sialic/Neuraminic acid, S.

**MC38-MOCK**				
**Possible composition**	**Registered Mass [*m/z*]$^+$**	**Calculated Mass [*m/z*]$^+$**	**Charge [H$^+$]**	**Average GU value**
Neutral				
H2N2	484.77	484.73	2	2.73
H3N2	565.77	565.76	2	3.22
H3N3	667.32	667.30	2	3.80
H3N4	768.83	768.84	2	4.33
H3N7	716.28	715.97	3	8.36
H4N2	646.81	646.78	2	4.03
H4N3	748.30	748.32	2	6.63
H4N4	849.83	849.86	2	5.22
H4N7	770.29	769.99	3	9.06

H5N2	727.88	727.81	2	5.01
H5N3	829.34	829.35	2	5.49
H5N4	620.94	620.93	3	6.01
H6N2	808.82	808.84	2	5.73
H6N3	607.25	607.25	3	6.38
H6N4	674.97	674.95	3	6.79
H6N7	878.31	878.03	3	7.58
H7N2	889.80	889.86	2	6.48
H7N3	661.29	661.27	3	7.27
H7N4	728.96	728.96	3	7.93
H7N5	796.65	796.66	3	8.24
H8N2	970.83	970.89	2	7.43
H9N2	1051.86	1051.92	2	8.36
H10N2	1132.90	1132.94	2	9.06
Fucosylated				
H2N2F1	557.77	557.76	2	2.79
H3N2F1	638.80	638.79	2	3.65
H3N3F1	740.33	740.33	2	4.21
H3N4F1	841.85	841.87	2	4.72
H3N5F1	629.28	629.27	3	5.37
H3N6F1	696.95	696.97	3	5.79
H4N2F1	719.95	719.81	2	6.30
H4N3F1	821.34	821.35	2	4.92
H4N4F1	922.87	922.89	2	5.49

H5N4F1	669.60	669.61	3	6.38
H6N4F1	723.64	723.63	3	7.21
H7N4F1	777.64	777.65	3	8.08
H7N5F1	845.53	845.34	3	9.77
H7N6F1	912.63	913.04	3	9.36
H8N5F1	899.31	899.36	3	9.23
H9N5F1	953.31	953.38	3	10.26
H4N6F2	799.65	799.67	3	11.59
Sialylated				
H3N4S1	610.39	609.93	3	4.66
H5N3S1	650.27	650.27	3	6.18
H5N4S1	717.97	717.96	3	6.75
H6N3S1	704.30	704.29	3	6.99
H6N4S1	771.98	771.98	3	7.43
H7N6S1	721.51	721.29	4	8.85
H4N4S2	761.30	760.97	3	9.36
H5N4S2	814.99	814.99	3	7.27
H6N5S2	702.34	702.78	4	10.96
H7N6S2	794.05	794.06	4	9.52
Mixed				
H4N3F1S1	966.85	966.89	2	5.62
H4N4F1S1	712.43	712.63	3	6.26
H4N5F1S1	585.39	585.49	4	11.34
H5N4F1S1	766.65	766.65	3	6.99

H6N4F1S1	820.66	820.66	3	7.77
H6N5F1S1	888.30	888.36	3	8.08
H7N6F1S1	757.44	757.80	4	9.43
H8N5F1S1	996.34	996.39	3	9.65
H5N4F1S2	863.66	863.68	3	7.58
H6N5F1S2	739.28	739.29	4	8.57
H7N5F1S2	779.54	779.81	4	9.52
H7N6F1S2	830.65	830.58	4	10.04
H6N5F1S3	811.70	812.07	4	9.43
H7N4F1S3	802.07	801.81	4	8.85

**MC38-FUT4**				
**Possible composition**	**Registered Mass [*m/z*]$^{+}$**	**Calculated Mass [*m/z*]$^{+}$**	**Charge [H^{+}]**	**Average GU value**
Neutral				
H2N2	484.76	484.73	2	2.71
H3N2	565.77	565.76	2	3.20
H3N3	667.32	667.30	2	3.78
H3N4	768.83	768.84	2	4.32
H3N6	648.39	648.28	3	9.29
H3N7	716.28	715.97	3	7.93
H4N2	646.80	646.78	2	4.02
H4N3	748.32	748.32	2	6.63

H4N4	849.83	849.86	2	5.21
H4N7	770.29	769.99	3	9.06
H5N2	727.82	727.81	2	4.57
H5N3	829.35	829.35	2	5.60
H5N4	620.93	620.93	3	6.00
H6N2	808.81	808.84	2	5.47
H6N3	910.36	910.38	2	6.37
H7N2	889.82	889.86	2	6.48
H7N3	661.28	661.27	3	7.26
H7N8	750.49	750.05	4	7.52
H8N2	970.84	970.89	2	7.66
H9N2	1051.91	1051.92	2	8.36
H9N8	830.32	831.08	4	11.64
Fucosylated				
H2N2F1	557.79	557.76	2	2.71
H3N2F1	638.82	638.79	2	3.64
H3N3F1	740.32	740.33	2	4.10
H3N4F1	841.85	841.87	2	4.71
H3N5F1	629.26	629.27	3	5.11
H3N6F1	696.96	696.97	3	5.73
H3N9F1	900.31	900.05	3	9.44
H4N2F1	719.95	719.81	2	6.31
H4N3F1	821.52	821.35	2	4.91
H4N4F1	922.86	922.89	2	5.47

H5N2F1	801.20	800.84	2	7.38
H5N3F1	601.90	601.92	3	6.24
H5N4F1	669.61	669.61	3	6.37
H5N5F1	737.32	737.31	3	6.98
H6N3F1	655.95	655.94	3	7.20
H6N4F1	723.64	723.63	3	7.26
H6N5F1	791.30	791.33	3	7.66
H7N4F1	777.64	777.65	3	7.38
H7N5F1	845.33	845.34	3	8.60
H7N6F1	912.65	913.04	3	8.85
H8N5F1	899.66	899.36	3	9.29
H3N3F2	813.36	813.35	2	4.91
H4N3F2	894.33	894.38	2	5.79
H4N4F2	995.94	995.92	2	6.24
H4N5F2	731.98	731.98	3	6.74
H5N4F2	717.96	718.30	3	6.63
H6N4F2	771.97	772.32	3	7.38
H6N5F2	839.71	840.01	3	9.81
H4N4F3	712.63	712.97	3	6.31
H4N5F3	780.64	780.66	3	7.93
H5N4F3	767.0	766.99	3	6.90
H5N5F3	834.31	834.68	3	7.52
H6N4F3	820.65	821.00	3	8.85
H6N5F3	888.32	888.69	3	8.24

Sialylated				
H4N3S1	894.33	893.87	2	5.73
H4N4S1	664.30	663.94	3	6.31
H4N5S1	731.98	731.64	3	6.63
H5N3S1	650.28	650.27	3	6.24
H5N4S1	717.96	717.96	3	6.48
H6N3S1	704.29	704.29	3	7.12
H6N4S1	771.97	771.98	3	7.47
H6N5S1	839.71	839.67	3	8.09
H4N5S2	828.32	828.67	3	8.85
H5N4S2	815.32	814.99	3	7.52
H5N5S2	882.30	882.69	3	9.81
H6N5S2	702.95	702.78	4	8.85
Mixed				
H4N4F1S1	712.64	712.63	3	6.21
H4N4F2S1	761.31	761.31	3	9.06
H4N5F1S1	585.48	585.49	4	7.93
H5N4F1S1	766.64	766.65	3	6.98
H5N4F2S1	815.32	815.33	3	7.52
H5N4F3S1	863.66	864.02	3	7.38
H5N5F1S1	834.31	834.34	3	8.85
H6N4F1S1	820.65	820.66	3	7.82
H6N5F1S1	888.32	888.36	3	8.50
H6N5F2S1	702.95	703.03	4	10.03

H5N4F1S2	863.66	863.68	3	7.66
H7N6F1S2	830.33	830.58	4	9.81
H7N4F1S3	802.26	801.81	4	9.29

**MC38-FUT9**				
**Possible composition**	**Registered Mass [*m/z*]$^+$**	**Calculated Mass [*m/z*]$^+$**	**Charge [H$^+$]**	**Average GU value**
Neutral				
H2N2	484.77	484.73	2	2.72
H3N2	565.78	565.76	2	3.22
H3N3	667.31	667.30	2	3.80
H3N4	768.84	768.84	2	4.33
H3N6	648.32	648.28	3	9.29
H3N7	716.41	715.97	3	7.93
H4N2	646.80	646.78	2	4.03
H4N3	748.33	748.32	2	6.64
H4N4	849.85	849.86	2	5.12
H4N7	770.28	769.99	3	8.86
H4N8	628.50	628.51	4	5.01
H5N2	727.82	727.81	2	4.58
H5N3	829.34	829.35	2	5.48
H5N4	620.94	620.93	3	6.01
H6N2	808.69	808.84	2	5.48

H6N4	674.95	674.95	3	6.91
H6N5	742.64	742.64	3	7.27
H7N2	889.84	889.86	2	6.64
H8N2	970.84	970.89	2	7.38
H8N7	740.27	739.80	4	11.66
H9N2	1051.86	1051.92	2	8.36
H9N3	769.29	769.31	3	9.44
H10N2	1132.89	1132.94	2	9.07
Fucosylated				
H2N2F1	557.77	557.76	2	2.79
H3N2F1	638.82	638.79	2	3.68
H3N3F1	740.33	740.33	2	4.21
H3N4F1	841.85	841.87	2	4.72
H3N5F1	629.26	629.27	3	5.12
H3N6F1	696.31	696.97	3	5.73
H4N2F1	719.33	719.81	2	6.32
H4N3F1	821.33	821.35	2	5.37
H4N4F1	922.84	922.89	2	5.48
H5N2F1	801.26	800.84	2	7.38
H5N3F1	601.91	601.92	3	6.01
H5N4F1	669.62	669.61	3	6.38
H6N3F1	655.94	655.94	3	7.19
H6N4F1	723.64	723.63	3	7.27
H6N5F1	791.31	791.33	3	7.67

H7N4F1	777.56	777.65	3	8.09
H7N5F1	845.32	845.34	4	8.61
H8N5F1	899.66	899.36	3	9.29
H3N3F2	813.36	813.35	2	4.92
H4N3F2	894.38	894.38	2	5.88
H4N4F2	995.91	995.92	2	6.32
H4N5F2	731.73	731.98	3	6.64
H5N4F2	717.97	718.30	3	6.99
H6N4F2	772.31	772.32	3	7.38
H6N5F2	839.65	840.01	3	9.81
H4N4F3	712.67	712.97	3	6.32
H4N5F3	586.19	585.75	4	6.64
H5N4F3	766.64	766.99	3	6.99
H6N4F3	820.65	821.00	3	8.86
H6N5F3	888.33	888.69	3	9.81
H7N6F3	757.78	758.06	4	9.44
Sialylated				
H4N3S1	893.98	893.87	2	5.80
H4N4S1	663.94	663.94	3	6.23
H4N5S1	731.73	731.64	3	6.64
H5N3S1	650.61	650.27	3	7.27
H5N4S1	717.97	717.96	3	6.64
H6N3S1	704.30	704.29	3	7.13
H6N4S1	772.31	771.98	3	7.45

H6N5S1	839.98	839.67	3	8.09
H4N5S2	828.32	828.67	3	8.86
H5N4S2	815.30	814.99	3	7.67
H6N5S2	936.67	936.70	3	8.86
H7N6S2	794.30	794.06	4	9.97
H6N5S3	775.66	775.55	4	9.29
Mixed				
H4N4F1S1	712.67	712.63	3	6.23
H4N4F2S1	761.32	761.31	3	9.07
H4N5F1S1	585.55	585.49	4	7.93
H5N4F1S1	766.64	766.65	3	7.13
H5N4F2S1	815.30	815.33	3	7.54
H6N4F1S1	820.99	820.66	3	7.75
H6N5F1S1	888.65	888.36	3	8.50
H6N5F2S1	936.26	937.04	3	9.07
H7N6F1S1	757.78	757.80	4	9.44
H5N4F3S2	960.85	961.05	3	9.44
H7N5F1S2	779.81	779.81	4	9.29
H7N6F1S2	830.35	830.58	4	10.47
H7N7F1S2	881.31	881.35	4	9.81
H7N4F1S3	802.24	801.81	4	9.97
H8N7F1S4	854.20	854.13	5	12.42

Supplementary Table III: Example of HILIC-(U)HPLC-FLR-ESI-MS/MS analysis with fragments of *m/z* 766.64 (possible composition H5N4F3) from the MC38-FUT9 cell line.

**MC38-FUT9**			
**Registered Mass [*m/z*]$^+$**	**Calculated Mass [*m/z*]$^+$**	**MS/MS Fragment**	**Intensity (%)**
366.06	366.14	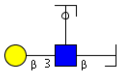	94.30
441.46	441.27	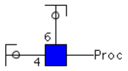	44.30
512.21	512.20	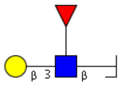	79.87
528.27	528.19	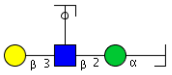	10.75
587.55	587.33	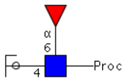	19.66
644.35	645.01	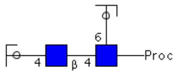	13.84
674.39	674.25	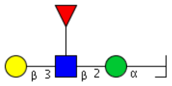	10.43
790.63	790.41	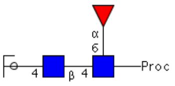	13.23

952.50	952.46	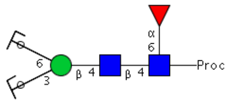	6.39
1039.33	1039.38	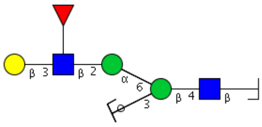	8.32
1114.61	1114.51	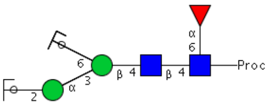	21.10
1201.35	1201.44	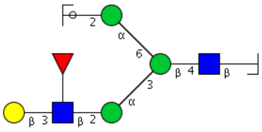	8.71
1276.34	1276.57	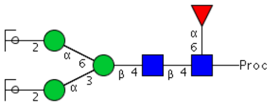	13.06
1347.37	1347.49	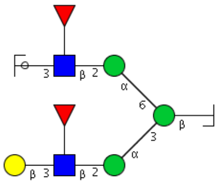	4.65
1641.75	1641.70	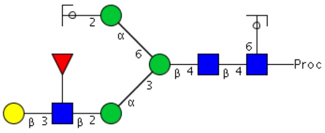	2.92
1787.88	1787.76	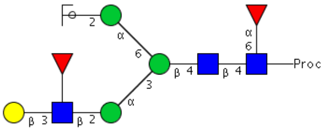	5.27
